# The mononuclear metal center of type-I dihydroorotase from aquifex aeolicus

**DOI:** 10.1186/1471-2091-14-36

**Published:** 2013-12-09

**Authors:** Brian FP Edwards, Roshini Fernando, Philip D Martin, Edward Grimley, Melissa Cordes, Asmita Vaishnav, Joseph S Brunzelle, Hedeel Guy Evans, David R Evans

**Affiliations:** 1 Department of Biochemistry and Molecular Biology, Wayne State University School of Medicine, 540 East Canfield Street, Detroit, MI 48201, USA; 2 Department of Chemistry, Eastern Michigan University, Ypsilanti, MI 48197, USA; 3 Life Sciences Collaborative Access Team, Northwestern University Center for Synchrotron Research, Argonne, IL 60439, USA

**Keywords:** Aspartate transcarbamoylase, Carbamoyl phosphate synthetase, CAD, Dihydrorotase, Metalloenzymes, Pyrimidine biosynthesis, Thermophile, Zinc ligands

## Abstract

**Background:**

Dihydroorotase (DHO) is a zinc metalloenzyme, although the number of active site zinc ions has been controversial. *E. coli* DHO was initially thought to have a mononuclear metal center, but the subsequent X-ray structure clearly showed two zinc ions, α and β, at the catalytic site. *Aquifex aeolicus* DHO, is a dodecamer comprised of six DHO and six aspartate transcarbamoylase (ATC) subunits. The isolated DHO monomer, which lacks catalytic activity, has an intact α-site and conserved β-site ligands, but the geometry of the second metal binding site is completely disrupted. However, the putative β-site is restored when the complex with ATC is formed and DHO activity is regained. Nevertheless, the X-ray structure of the complex revealed a single zinc ion at the active site. The structure of DHO from the pathogenic organism, *S. aureus* showed that it also has a single active site metal ion.

**Results:**

Zinc analysis showed that the enzyme has one zinc/DHO subunit and the addition of excess metal ion did not stimulate catalytic activity, nor alter the kinetic parameters. The metal free apoenzyme was inactive, but the full activity was restored upon the addition of one equivalent of Zn^2+^ or Co^2+^. Moreover, deletion of the β-site by replacing the His180 and His232 with alanine had no effect on catalysis in the presence or absence of excess zinc. The 2.2 Å structure of the double mutant confirmed that the β-site was eliminated but that the active site remained otherwise intact.

**Conclusions:**

Thus, kinetically competent *A. aeolicus* DHO has a mononuclear metal center. In contrast, elimination of the putative second metal binding site in amidohydrolyases with a binuclear metal center, resulted in the abolition of catalytic activity. The number of active site metal ions may be a consideration in the design of inhibitors that selectively target either the mononuclear or binuclear enzymes.

## Background

**Figure 1:**
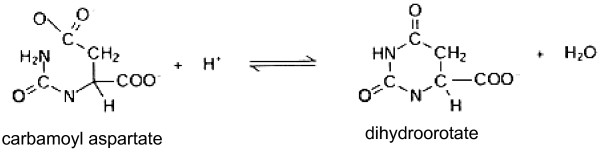
The reaction catalyzed by DHO.

Dihydroorotase (DHO) catalyzes the reversible interconversion of carbamoyl aspartate and dihydroorotate, the third step in *de novo* pyrimidine biosynthesis. The protein is a member of the amidohydrolase family of enzymes [1] Figure 1.

The same reaction occurs in nearly all organisms but the enzymes are very diverse. A phylogenetic analysis [2] demonstrated that DHOs belong to two distinct classes that differ in sequence, size and oligomeric structure. Type-I enzymes, which evolved first, are larger, 43–45 kDa, and are often associated with aspartate transcarbamoylase (ATC), the enzyme that catalyzes the formation of carbamoyl aspartate, the substrate for DHO. Type-II DHOs, which evolved more recently, contain only the catalytic core (38 kDa) and typically have 50 and 10 residues deleted from the amino and carboxyl ends, respectively, as well as several internal insertions and deletions compared to the type-I enzymes.

**Figure 2:**
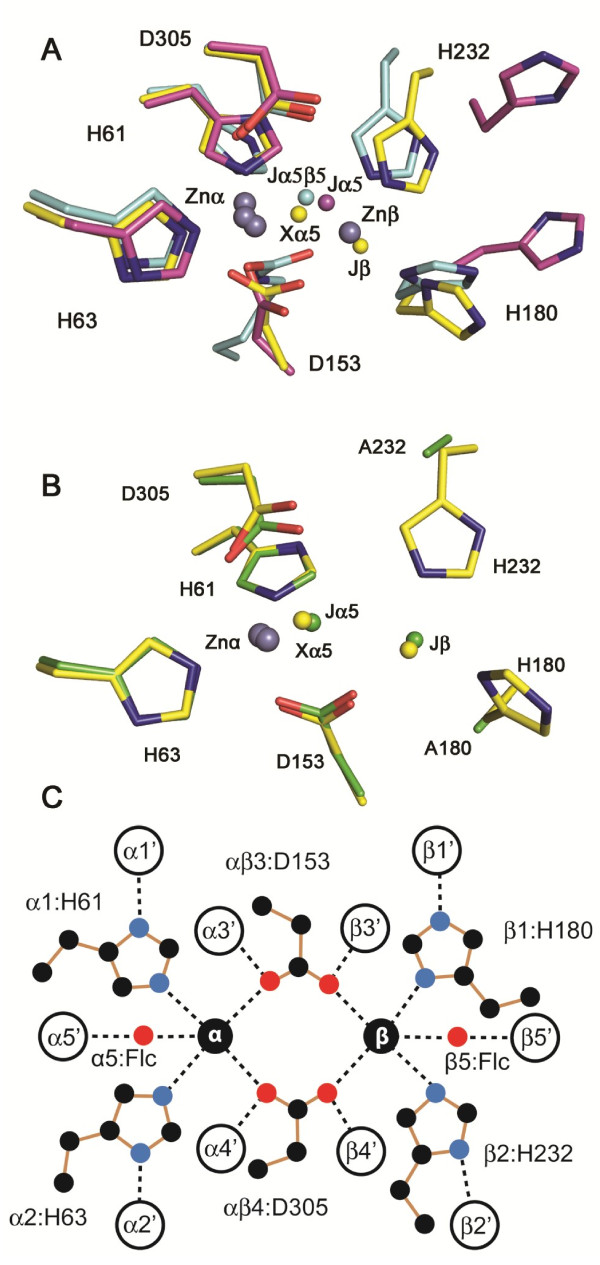
**The DHO Active Sites. (A)** The active sites of DHO from *E. coli* (pdb:1j79A; cyan carbon atoms), *A. aeolicus* (pdb:1xrfA; magenta carbon atoms), and the *A. aeolicus* native complex of DHO and ATC (pdb:3d6nA; yellow carbon atoms). Zinc atoms are shown as grey spheres. Significant water molecules (“J”) and ligand atoms (“X”) are also differentiated by the carbon color. The water molecules are labeled by their role in the active site (Figure 2C) because the numbers assigned to water molecules in the same site vary among the numerous DHO structures. Here, Jα5β5 is HOH1407 in 1j79.pdb; Jα5 is HOH2 in 1xrf.pdb; Xα5 is the OG1 atom from the citrate ligand in 3d6n.pdb; and Jβ is HOH577 in 3d6n.pdb; **(B)** Superposition of the active sites of *A. aeolicus* DHO from the native complex (3d6n.pdb); yellow carbon atoms) and the mutant complex (4bjh.pdb; green carbon atoms). Zinc atoms are shown as grey spheres. Significant water molecules (“J”) and ligand atoms (“X”) are differentiated by the carbon color. Jα5 is HOH2018/A in 4bjh.pdb; Xα5 is the OG1 atom from the citrate ligand in 3d6n.pdb; and Jβ is HOH577 in the wild-type 3d6n.pdb and HOH2080/A in 4bjh.pdb. **(C)** A schematic diagram of the first and second binding shells for two zinc atoms in the active sites of type-I and type-II DHO enzymes. The first shell ligands are shown as labeled residues for *A. aeolicus* DHO. The second shell ligands are shown as labeled circles. The analogous first shell residues for *S. aureus*, *B. anthracis*, and *T. thermophilus* DHO and the second shell ligands for all four species are listed in the supplement (Additional file 1: Table S1) together with values for the distances marked by dashed lines in the diagram.

*E. coli* DHO, a type-II enzyme, is an eight stranded β- or TIM barrel, a structural organization typical of amidohydrolyases [3]. While this enzyme was initially reported to have a mononuclear metal center [4], the X-ray structure showed that there are two zinc ions at the active site (Figure 2A) bridged by a water molecule and a carboxylated lysine (Lys 102). The zinc in the α-site has a distorted trigonal bipyramidal coordination sphere and binds two histidines, an aspartate, the carboxylated lysine and the bridging solvent. The zinc in the β-site is more solvent accessible and has a distorted tetrahedral coordination sphere consisting of two histidines, the carboxylated lysine and the bridging hydroxide. A catalytic mechanism was proposed based on the presence of two zinc ions and the juxtaposition of residues near the bound substrates [3].

We have cloned and expressed *Aquifex aeolicus* DHO, a type-I enzyme in *E. coli*[5,6]. The recombinant protein, with a molecular mass of 43 kDa, was expressed at high levels and was soluble but lacked catalytic activity. The enzyme was monomeric and contained a single zinc ion. However, it formed an active complex with *A. aeolicus* ATC consisting of six copies of each type of subunit that had both ATC and DHO activities [6]. The structure of the isolated DHO subunit, determined in two space groups is comprised of two domains, a distorted TIM barrel that resembles the structure of *E. coli* DHO and a composite domain formed by residues 1–55 at the amino end and 366–422 at the carboxyl end of the polypeptide [7] that is found in many metal-dependent hydrolases [8-11].

The two *A. aeolicus* DHO structures suggested several possibilities that could account for the lack of activity of the isolated subunit. A water molecule was bound to the zinc in one X-ray structure (1xrf.pdb), while in the other structure (1xrt.pdb), Cys181 in a loop, designated loop A, was ligated to the active site zinc, occluding the active site and blocking access of the substrates. Most of loop A was disordered and invisible in the electron density map, as were two other loops, B and C. The zinc ion was bound to a site corresponding to the less solvent accessible α-site in the *E. coli* enzyme. The zinc ligands were His61, His63, Asp153, and (Jα5) or Cys181 in an approximately tetrahedral arrangement (Figure 2A). The equivalent ligands for the zinc β-site in *E. coli* DHO were conserved but disarranged. Asp153 was close to the analogous carboxylysine in *E. coli* DHO but the two equivalent histidine ligands, His180 ND1 and His232 NE2 (*A. aeolicus* numbering) were rotated out of the active site by 7.4 Å and 7.1 Å, respectively, from the putative zinc β-position.

The X-ray structure of the active *A. aeolicus* DHO-ATC complex (3d6n.pdb) showed that all of the disordered loops now had a well-defined conformation and that an extensive movement of loop A had displaced it from the active site [12]. The zinc in the α-site now had tetrahedral geometry with His61, His63, Asp153, and one oxygen from citrate, a substrate analog, as ligands, with an average bond distance of 2.2 Å. The next closest ligand for trigonal bipyramidal coordination was Asp305 at 2.7 Å. Moreover, the β-zinc binding site was fully reconstituted when the DHO and ATC subunits associate to form the active dodecamer. However, no zinc was bound to the putative β-site, instead a water molecule, HOH577, (Jβ in Figure 2A and B) occupied this position. As a consequence, one of the carbonyl oxygens of Asp153 that was formerly bound to the N of Gly154 now formed a hydrogen bond (2.6 Å) with the water molecule in the putative β-site. In addition, His180 and His232 rotate into the active site forming tight hydrogen bonds (His180 ND1: 2.2 Å; His232 NE2: 2.4 Å) with the same water molecule upon formation of the DHO-ATC complex (Figure 2A and B). Consequently, the positions of the zinc ligands were virtually the same as the corresponding β-zinc ligands in the *E. coli* enzyme (Figure 2C).

In mammals, dihydroorotase is a domain of a large multifunctional protein called CAD that also catalyzes the ATC and glutamine dependent carbamoyl phosphate synthetase activities [13,14]. It was previously found that mammalian DHO has a single active site zinc [15-17]. However, recent structural studies carried out by Santiago Ramon-Maiques (Spanish National Cancer Research Centre (CNIO), Madrid, Spain) showed that the DHO domain of human CAD, like the *E. coli* enzyme, has two zincs at the active site^a^.

Several observations have led many investigators to believe that the active site of all DHOs are binuclear, 1) the β-Zn site ligands are universally conserved, 2) these residues are located in the correct position (as in *A. aeolicus* DHO) with an appropriate orientation to bind a second zinc, 3) the β-site is significantly more accessible to solvent, so that the second zinc may have been lost during protein purification or crystallization, 4) zinc analysis was typically preceded by dialysis to remove adventitiously bound metal [4,14,15] that may have resulted in the loss of the second metal ion and 5) both *E. coli* and mammalian DHO, which were initially found to have one zinc, were subsequently shown to have two metal ions.

The current study was undertaken to determine whether the lack of a second metal ion in *A. aeolicus* DHO is an artifact or whether the reversible condensation of carbamoyl aspartate to form dihydroorotate can be catalyzed by a mononuclear metal center.

## Results

### Metal analysis of the DHO-ATC complex and subunits

Zinc analysis by ICP-mass spectrometry confirmed the results of previous studies [6,7] that indicated that the purified, isolated DHO subunit has only one mole of zinc per mole of protein (Table 1). The analysis also showed that the protein had no detectable bound cobalt, another metal ion that can support DHO catalysis. The growth media for the over-expression of ATC and DHO subunits was usually not supplemented with metals, so one possibility is that the β-site may have been unoccupied because the availability of the metal ion was limiting. To test this possibility, the growth media was supplemented with 100 μM or 1 mM ZnCl_2_. The protein isolated from these different growth media (Table 1) also had one zinc atom and no cobalt, indicating that the unsupplemented media contained sufficient metal ions to saturate the protein metal binding sites. Similarly, the addition of Zn^2+^ or Co^2+^ to the purified DHO subunit did not significantly increase the incorporation of either metal ion (Table 2).

**Table 1:** **Metal analysis**^**a**^**of DHO expressed with media supplemented with metal ions**

**Protein**	**Growth Media**	**Molar ratio (M**^**2+**^**/protein)**	
	**ZnCl**_**2**_**(mM)**	**Zn**^**2+**^	**Co**^**2+**^
DHO	0	0.88	0.001
	0.10	1.21	0.000
	1.00	0.97	0.000

^a^ICP mass spectrometry.

**Table 2:** **Metal analysis**^**a**^**of purified DHO incubated with metal ions**

**Protein**	**ZnCl**_**2**_	**CoCl**_**2**_	**Molar ratio**	**(M**^**2+**^**/protein)**
	**mM**	**mM**	**Zn**^**2+**^	**Co**^**2+**^
DHO	0	0	0.88	0.001
	0.10	0	1.21	0.000
	1.00	0	0.97	0.000
	0	0.10	1.15	0.123

^a^ICP mass spectrometry.

The zinc content of the DHO subunit and the DHO-ATC complex was also determined by a colorimetric 4-(2-Pyridylazo) resorcinol (PAR) assay (*Methods*). The analysis (Table 3) was conducted at 25°C, the temperature at which the crystals were grown, and at 75°C, the temperature at which the enzymes were assayed. Analysis of the ATC subunit showed that it contained no detectable zinc. The DHO subunit had about 0.84 equivalents of zinc at 25°C and 75°C indicating that the metal binding α-site was not completely occupied. The DHO subunit was then incubated with a 5-fold stoichiometric excess zinc, followed by removal of the excess metal by spin column chromatography. Zinc analysis indicated that the zinc content increased to 0.97-1.07, consistent with the interpretation that there was a small loss of zinc during the purification of protein. The presence of EDTA did not significantly alter the zinc content of the isolated DHO subunit.

**Table 3:** **Zinc analysis**^**a**^**of wild type and mutant proteins**

**Protein**	**EDTA**	**Zn**	**Zn/DHO**	
	*μM*	*molar ratio*	*25°C*	*75°C*
ATC	0	0	0	0
*Wild Type DHO*				
DHO	0	0	0.84	0.83
DHO	0	5X	1.07	0.97
DHO	100	5X	0.98	1.01
DHO-ATC	0	0	1.06	nd
DHO-ATC	0	5X	0.98	0.95
DHO-ATC	100	5X	0.95	0.98
*DHO Double Mutant*				
DHO	0	0	0.71	0.90
DHO	0	5X	0.94	1.27
DHO	100	5X	0.98	1.30
DHO-ATC	0	0	1.20	nd
DHO-ATC	0	5X	0.93	1.14
DHO-ATC	100	5X	0.95	1.29

^a^Spectrophotometric analysis.

A similar series of experiments was carried out on the DHO-ATC complex. This analysis (Table 3) indicated that the protein contained slightly less than one equivalent of zinc per DHO subunit, a value that increased to give approximately one-to-one stoichiometry when excess zinc was provided. Again, the zinc content was not appreciably altered by the addition of EDTA.

### Titration of DHO-ATC complex with zinc and cobalt

**Figure 3:**
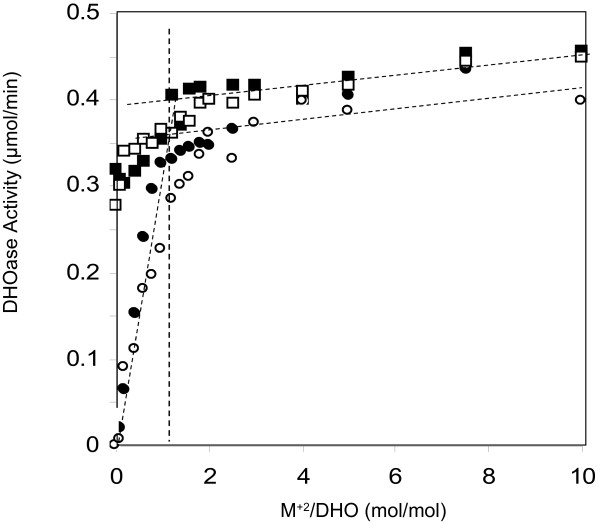
**Titration of DHO-ATC and apo-DHO-ATC with zinc and cobalt.** The DHO-ATC complex in 0.05 mM TrisHCl, pH 7.4 was titrated with zinc chloride (■) and with cobalt chloride (□) and the catalytic activity was assayed with a dihydroorotate concentration of 8 mM at 72°C. The metal ion was removed from the DHO subunit by dialysis against pyridine-2, 6-dicarboxylate and the DHO-ATC complex was reconstituted (Methods). The apoezyme was then titrated with zinc (●) and with cobalt (□) and assayed.

To determine whether additional exogenous metal ions had an effect on the catalytic activity of the DHO-ATC, the complex was titrated with metal ions (Figure 3). There was a small activation of the DHO, approximately 20%, as the molar ratio of zinc ion to protein increased, a further indication that the α-site was not fully occupied. A similar result was obtained when the complex was titrated with cobalt chloride.

The endogenous zinc ion was extracted from the DHO subunit by exhaustive dialysis against the strong chelating agent, pyridine-2, 6-dicarboxylate (Methods). The complete removal of the metal was confirmed by zinc analysis. The metal free apoenzyme had no catalytic activity and was then titrated with zinc chloride and cobalt chloride. The endpoint with both metal ions corresponded to a molar ratio of the metal ion to protein of one. There was only a slight increase in catalytic activity with increasing in zinc beyond the endpoint of the titration curve. The titration curves for zinc chloride and cobalt chloride were almost identical, indicating that both metal ions are equally effective in promoting catalysis.

### Effect of excess zinc on the steady state kinetics of DHO-ATC

**Figure 4:**
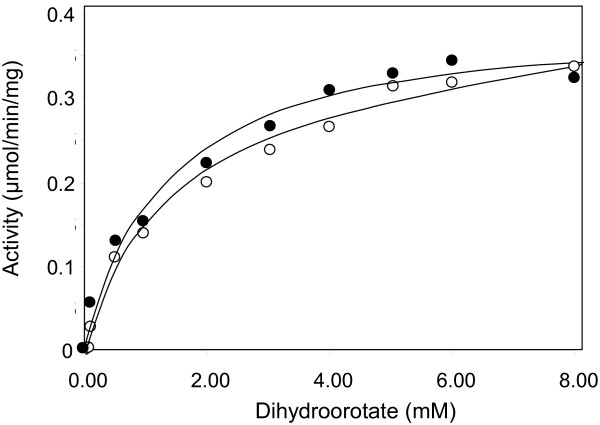
**Effect of excess zinc on the DHO-ATC steady state kinetics.** A dihydroorotate saturation curve of DHO-ATC (Methods; 250 mM Tris acetate, pH 8.3, 72°C) was carried out in the presence of a 5-fold molar excess of ZnCl_2_ (○) or in the absence of exogenous zinc (●). The kinetic parameters are summarized in Table 4.

Although additional zinc had no apparent effect on the activity at near saturating concentrations of the substrate, excess metal ion may alter the steady state kinetic parameters. The dihydroorotate saturation curve for the DHO-ATC complex at 75°C (Figure 4, Table 4) gave K_m_ value of 1.52 ± 0.30 mM and a V_max_ of 0.250 ± 0.017 μmol/min. The corresponding values for the complex assayed in the presence of a 5-fold molar excess of zinc gave K_m_ and V_max_ values of 1.93 ± 0.35 mM and 0.270 ± 0.018 μmol/min, respectively. Thus, there was little effect of additional metal ions on DHO catalysis or substrate binding.

**Table 4:** **Steady state kinetic parameters of the wild type and mutant proteins**^**a**^

**Protein**	**K**_**m**_	**V**_**max**_
	*mM*	*μmol/min*
Wild Type	1.52 ± 0.30	0.250 ± 0.017
Wild Type + 5-fold ZnCl_2_	1.93 ± 0.35	0.270 ± 0.018
Wild Type	1.03 ± 0.16	0.233 ± 0.011
His180Ala	1.65 ± 0.26	0.238 ± 0.013
His232Ala	1.34 ± 0.22	0.224 ± 0.012
His180Ala/His232Ala	1.55 ± 0.30	0.186 ± 0.012

^a^The dihydroorotate saturation curves (Figures 4 and 7) were fit by a least squares analysis to the Michaelis Menten equation using the program KaleidaGraph.

### X-ray structure of the β-site mutants^b^

Two residues, His180 and His232, that bind the second zinc ion in the β-site (Figure 2) of type-I DHO (*T. thermophilus*, 2z00.pdb; *B. anthracis*, 3mpg.pdb) and type-II enzymes (*E. coli*, 1j79.pdb; *C. jejuni* (3pnu.pdb); *S. tymphurium*, 3jze.pdb) and interact with a water molecule in *A. aeolicus* DHO (3d6n.pdb), were replaced with alanine by site-directed mutagenesis. The single and the double His180Ala/His232Ala were constructed. The double mutant was crystallized under similar conditions as the wild type enzyme [12] except that citrate, which binds weakly to the DHO and ATC active sites, was omitted. Both the DHO substrate, dihydroorotate, and the ATC bisubstrate analogue, PALA, were included in the “cryo” solution in an attempt to increase the order and thus the X-ray diffraction of the crystals. However, after preliminary refinements at higher resolutions, the data were truncated to 2.2 Å, 0.1 Å better than the native complex, at a conservative value of 2.3 for I/σ in the last shell. The final R-factor, using TLS refinement, is 0.159 and the final R_free_ is 0.201.

**Figure 5:**
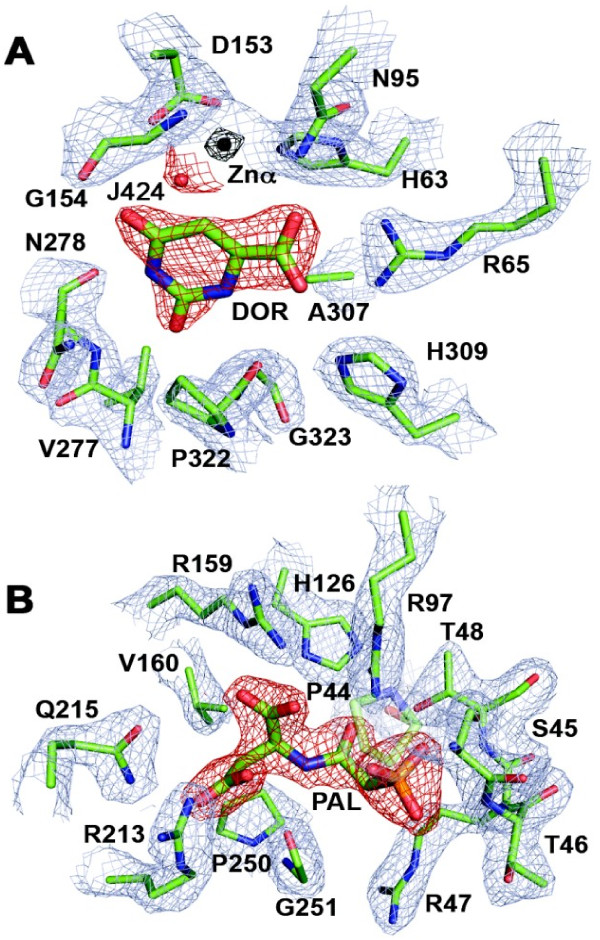
**Electron density, contoured at 2 sigma, for the DHO and ATC active sites in the mutant complex. (A)** the “omit density” is shown for the substrate dihydroorotate (DOR, red mesh), the zinc atom (Znα, black mesh), the catalytic water molecule (Jα5 or in the text, HOH2018/A, red mesh) and the 2Fo-Fc electron density of residues within 4 Å of the substrate (blue mesh). Only side chains are shown except for residues P322, G323, V277, N278, and G154 which have main chain contacts with dihydroorotate (DOR425) atoms. Five other water molecules within 4 Å of DOR have been omitted for clarity. **(B)** The “omit density” for PALA (PAL, red mesh) in the ATC active site and the 2Fo-Fc electron density is shown for all ATC residues within 4 Å of the PALA molecule (blue mesh). Only side chains are shown except for residues P44-T48 and G251, which have main chain contacts with PALA atoms. P44 and its electron density, which overlay the inhibitor in this view, are shown at 50% transparency. Six water molecules within 4 Å of PALA have been omitted for clarity.

**Figure 6:**
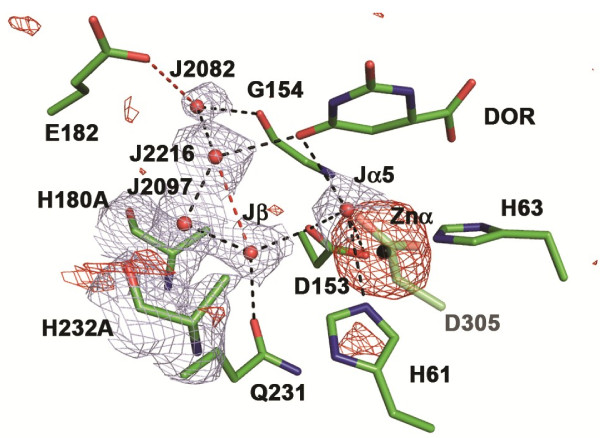
**Water structure and anomalous difference density in the active site of the double mutant.** The 2Fo-Fc electron density (blue mesh) is shown for the 5 water molecules contoured at 1 sigma and the two mutated alanine residues contoured at 1.5 sigma. When the anomalous difference density map was contoured at 3 sigma for the entire active site, strong difference density was observed only at the α-zinc atom (black sphere, red mesh) with a few noise contours near H61 and H232A. In particular, there was no anomalous difference density at Jβ, the putative β-metal site.

The asymmetric unit in space group H23 includes one DHO subunit with one zinc atom and dihydroorotate in its active site, one ATC subunit with PALA in its active site, three barium atoms bound to the ATC subunit, and twelve ethylene glycol molecules (PDB ID: edo). The barium ions, which increase the reproducibility, size, and diffraction of the crystals, have anomalous difference density peaks at 25, 15 and 7.0 sigma and are bound to the ATC subunit, which makes the sole crystal contact in the unit cell. The barium ions are quickly lost if omitted from the “cryo” solution, as was done with the native structure [12]. In this structure (Figure 5), the biological unit in the crystal is the same as the native structure, a tightly bound dodecamer with 23 symmetry formed by six asymmetric units. The full set of X-ray structure parameters are given in Table 5. The crystal structure of the double mutant showed strong anomalous difference density for zinc at the alpha site (above 17 sigma) and none at the beta site at the 3σ contour level (Figure 6). Consequently, Zn423 at the alpha site was refined at full occupancy and a water molecule assigned to the A-chain, HOH2080/A, was placed at the putative zinc beta site (Jβ in Figure 2B and Figure 6).

**Table 5:** Crystal structure statistics

**Statistics**	**Mutant**
PDB code	4BJH
Crystals	1
Space group	H32
Unit cell dimensions	
a (Å)	157.15
b (Å)	157.15
c (Å)	233.24
α (degrees)	90
β (degrees)	90
γ (degrees)	120
Chains per asymmetric unit	1 DHO, 1 ATC
Data collection	
Resolution (Å)	2.20
Reflections used	53365
Completeness (%)	99.4 (100.0)
Average I/σ	16.2 (2.3)
R_merge_ (%)	10.3 (75.6)
Refinement	
R factor (%)	16.1 (24.3)
R free (%)	20.3 (29.3)
Average B factor (Å2)	41.3
Atoms in asymmetric unit	6151
Number of water molecules	445
Rms bonds (Å)	0.016
Rms angle (degree)	1.558
Rms chiral	0.104

### Properties of the β-site mutants

In the absence of exogenous zinc, the stoichiometry of metal binding to the double mutant (Table 3) exhibited somewhat less than one to one stoichiometry. The molar ratio of Zn/protein was 0.71 – 0.90 for both the isolated DHO subunit and the DHO-ATC complex. As observed with the wild type enzyme, the occupancy increased to approximately 100% in the presence of a 5-fold molar excess of zinc. The zinc titration of the His180Ala and His232Ala mutants resembled that of the wild type enzyme in that the catalytic activity increased approximately 20% in the presence of exogenous zinc, presumably the result of filling the incompletely occupied σ-zinc site. In contrast, there was only a modest increase in the activity of the double mutant with increasing exogenous zinc.

**Figure 7:**
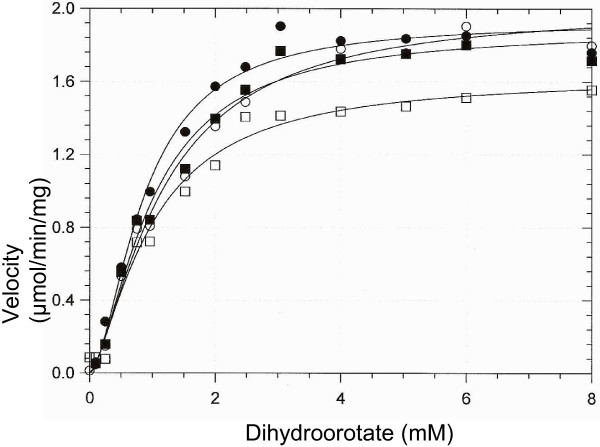
**Steady state kinetics of the wild type and β-site mutants.** A dihydroorotate saturation curve of DHO-ATC assayed as described in the Methods (250 mM Tris acetate, pH 8.3 at 72°C) was determined for the wild type (●), His180Ala (○), His232Ala (■) and the His180Ala/His232 (□). The kinetic parameters are summarized in Table 4.

Dihydroorotate saturation curves of the DHO-ATC complex reconstituted with the DHO mutants and native ATC (Figure 7) indicated that the steady state kinetic parameters (Table 4) of the wild type, His180Ala and His232Ala mutants were very similar. The K_m_ of the double mutant was unaltered (Table 4) but there was a small (21%) but significant decrease in the V_max_.

## Discussion

Metal analysis, the X-ray structure [6,7,12] and anomalous scattering difference maps showed that the isolated, inactive *A. aeolicus* DHO subunit has a single, functional zinc-binding site. However, the extensive reorganization of the DHO structure that occurs upon formation of a complex with ATC, results in the generation of a catalytically active protein with an intact putative second metal binding site (Figure 2). Thus, the possibility that a zinc ion was lost during crystallization raises the question that a second, more loosely bound zinc ion could be required for activity. The initial metal analysis of *E. coli* and human DHO revealed a single active site metal ion, but both enzymes were subsequently found to have a binuclear metal center, an observation that lends credence to this hypothesis. The consensus is emerging that all dihydroorotases are binuclear. The distinction could be important as different strategies could be envisioned in the design of drugs that target DHO depending on whether the active site has one or two metal ions. Differences in the metal center might be exploited to distinguish, for example, between human and some pathogenic DHOs.

To test the possibility that the *A. aeolicus* DHO may require a second zinc ion, the isolated DHO-ATC complex was titrated with zinc and the catalytic activity was assayed. The observed small increase in catalytic activity could be explained if the α-site metal ion occupancy was somewhat less than 100%. This interpretation was confirmed by zinc analysis of the isolated DHO subunit and the DHO-ATC complex, which showed that the occupancy of the isolated DHO subunit from which the dodecamer was reconstituted was 0.84 zinc/mol of subunit. Similarly, the steady state kinetic parameters were unaltered in the presence of a five-fold molar excess of zinc.

The most convincing evidence demonstrating that a second zinc ion is not required for catalysis was the replacement of two β-site first shell ligands, His180 and His232, with alanine, which eliminated the β-site but had no significant effect on the steady state kinetic parameters. In contrast, the replacement of the corresponding histidines in the β site of *Klebsiella pneumoniae* dihydroorotase [18] and allantoinase [19], both of which have a binuclear metal site, completely abolished catalytic activity. The crystal structure of the active double mutant showed a zinc atom in the α-site and a water molecule Jβ (HOH2080/A) near the β-site (Figure 2B, Figure 6) - thus eliminating the unlikely possibility that the one zinc atom was randomly distributed between the two sites. The observation that the elimination of the β-site ligands of the binuclear metal site enzymes abolishes catalytic activity, whereas there is no effect if the comparable ligands in the mononuclear enzyme are replaced, reinforces the argument that the two types of enzymes are distinct.

An alternate possibility should be considered that perhaps all dihydroorotases utilize a single zinc atom for catalysis and that the second zinc serves another purpose, perhaps enhancing the stability of the protein. This idea is countered by the mutagenesis studies, although it could be argued that the β-site ligands have an alternate unknown role in catalysis. However, we believe that this hypothesis is unlikely to be true as a plausible two-metal mechanism for *E. coli* DHO has been proposed based on the structure [3] that is well supported by subsequent biochemical [20] and quantum mechanical studies [21]. We have recently undertaken a molecular dynamics/quantum mechanical investigation based on the structure of the *A. aeolicus* DHO-ATC complex [12] that suggests that a one-zinc DHO can readily catalyze the reaction by an analogous, albeit somewhat different, mechanism. As discussed below, there is ample precedence for amidohydrolases catalyzing the same reaction having either one or two zinc ions depending on the organism.

### Mechanistic considerations

The structure of the double mutant enzyme offers other insights that are potentially relevant to the mechanism of the native enzyme. First, the catalytic water molecule, which is bound to the two zincs as Jα5β5 in the *E. coli* enzyme (Figure 2A) and the one zinc as Jα5 in *A. aeolicus* DHO (1rtf.pdb; Figure 2A) is retained in the mutant structure (Jα5: Figure 2B:, Figure 6), where it is poised, as in the *E. coli* complex with dihydroorotate (1j79A.pdb), to attack and cleave the ring forming carbamoyl aspartate. In the substrate complex with carbamoyl aspartate (1j79B.pdb, not shown) and the pseudo-substrate complex with citrate (3d6nA.pdb; Figure 2B), an oxygen atom of the ligand occupies the catalytic water site and binds directly to the zinc atom.

A second notable observation is the retention of a water molecule, HOH2080/A (Jβ:, Figure 2B, Figure 6) in the approximate location of the Znβ site in the structure of the mutant DHO despite the elimination of His180 and His232 that hydrogen bonds to the equivalent Jβ water molecule, HOH577, in the native protein complex (3d6nA.pdb, Figure 2B). In the mutant DHO, the space normally occupied by the imidazole rings of His180 and His232 is now occupied, respectively, by HOH77 and HOH280, which are also hydrogen bonded with the β-site water, HOH426 (Figure 6). Extensive theoretical calculations currently underway suggest that this second catalytic water molecule can significantly lower the activation energy of the reaction.

Although the mutant *A. aeolicus* DHO has alanine replacing the two histidines and dihydroorotate replacing citrate, it is still very similar to the native structure as evidenced by a rmsd value of 0.29 Å for the 422 Cα atoms. The two largest Cα displacements - Met1 (2.8 Å) and Pro41 (1.7 Å) - occur in the composite domain, which has higher B-values and worse density than the TIM-barrel catalytic domain, for which all the Cα displacements are less than one angstrom. This modest sensitivity to the ligand in the active site contrasts strongly with *E. coli* DHO, in which a mobile loop (Pro105 - Gly115) is hydrogen bonded to the substrate, carbamoyl aspartate, via residues Thr109 and Thr110 but flipped out of the active site when the product, dihydroorotate, is present (1ekx.pdb) [22]. The α-carbons of the two threonines are shifted by approximately 12 Å. When the mutant DHO is superposed using SSM onto the *E. coli* DHO subunit containing dihydroorotate (1ekxA.pdb), Met158 and Asp159 align structurally with Asn107 and Ser118, respectively in the *E. coli* subunit. In other words, SSM identifies the mobile loop in *E. coli* DHO, as a ten residue insert compared to the structure of *A. aeolicus* DHO. Interestingly, when the mutant DHO structure is superposed onto the *E. coli* DHO subunit containing carbamoyl aspartate (1ekxB.pdb), there are no *A. aeolicus* residues equivalent to Thr109 and Thr110 to hydrogen bond to the substrate. However, this observation must be verified by determining the structure of *A. aeolicus* DHO with the carbamoyl aspartate substrate in its active site.

### ATC conformational changes induced by PALA

The mutant DHO-ATC structure is the first with the bisubstrate analog, N-phosphoacetyl L-aspartate (PALA) bound to the active site of the *A. aeolicus* ATC subunit (Figure 5B). The PALA-ATC subunit superposes onto the native structure with an rmsd value of 0.71 Å for 291 residues and exhibits two major conformational changes triggered by PALA replacing citrate in the active site. The residues from Ser67 to Glu77 have Cα displacements above 1 Å with a maximum of 7.4 Å at Ser72. Together, Ser72 and Lys75 in this segment form hydrogen bonds - five in the mutant structure - directly to the PALA in the adjacent catalytic subunit, as first reported for *E. coli* ATC [23]. The second major conformational change involves segment Arg213 through Asn222, which has a maximum Cα displacement of 8.5 Å at Gln219. Arg213 and Gln215 form hydrogen bonds directly with the C5 carboxyl group of PALA. The large displacement of Gln219, which has no significant interactions with PALA or other residues, is a consequence, and not a cause, of the conformational change. An adjacent residue, Arg218, which has a Cα displacement of 7.8 Å, interacts indirectly with PALA through a hydrogen bond network with the PALA ligands Gln215, and Arg159.

In *E. coli* ATC, PALA binding triggers the transition from the T state to the active R state. Although the two largest conformational changes induced by PALA are “homologous” in the *A. aeolicus* and *E. coli* ATC, the former is much less plastic. The mutant *A. aeolicus* ATC has only 27 out of 291 residues with a Cα displacement greater than 1.0 Å relative to the native structure whereas the *E. coli* enzyme has 109 out of 310 residues that move over 1.0 Å due to PALA binding. This difference in conformational flexibility might be due to the native *A. aeolicus* structure having citrate bound in the PALA site, so it is already partially in the R-state, or to the *A. aeolicus* ATC being part of a larger complex that inhibits widespread conformational changes.

*E. coli* ATC, which is a complex of two trimers of catalytic chains and three dimers of regulatory chains, is notable for its large quaternary changes associated with the T to R transition upon PALA binding. The hetero dodecamer expands by 12 Å and the two trimers rotate by 10 degrees relative to each other [23]. However, PALA binding to the *A. aeolicus* mutant dodecamer, which is not an allosteric enzyme, has only a very modest effect. The “top” and “bottom” ATC trimers in the mutant dodecamer generated from crystallographic symmetry, when compared to the native complex, have rotated less than 2 degrees relative to each other and expanded by less than 0.5 Å.

### Comparison of amidohydrolase mono and dinuclear metal centers

**Figure 8:**
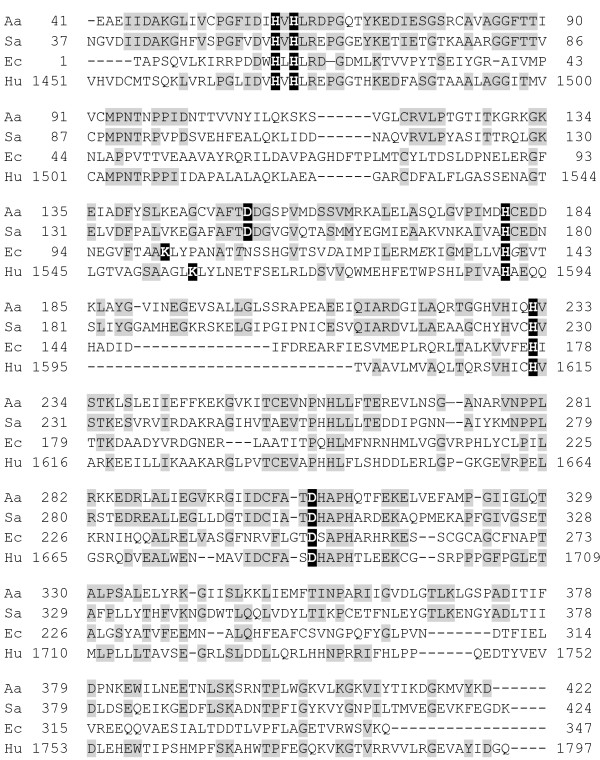
**DHO multiple sequence alignment.** The amino acid sequence of *A. aeolicus* (Aa), *S. aureus* (Sa), *E. coli* (Ec) and the DHO domain of human CAD (Hu) were aligned using Clustral W (ExPays Proteomics Site). The *A. aeolicus* residues that are conserved in *S. aureus*, *E. coli* and CAD are shaded gray. The α- and β-site Zn ligands are shown in white lettering on a black background. All of these residues are conserved and aligned with the exception of the active site ligand Asp153 in *A. aeolicus*. This residue is conserved in *S. aureus* DHO, but in *E. coli* and CAD, it is a Lys (Lys102 in *E. coli*), which is carboxylated and bridges the two zinc ions. The Lys is shifted upstream relative to the Asp in *A. aeolicus* and *S. aureus*. In a previous pair wise alignment [3] this lysine occupies the same position in the *E. coli* and CAD sequences.

Sequence alignment (Figure 8) shows that the one-zinc DHOs from *A. aeolicus* and *S. aureus* exhibit the highest sequence similarity (41% identity and 59% similarity) to each other. The *A. aeolicus* enzyme is much less similar to either of the two zinc enzymes, *E. coli* (23% identity, 39% similarity) or the mammalian enzyme (30% identity, 46% similarity). The differences in sequence per se cannot explain the differences between the one and two zinc enzymes, but suggest a closer phylogenetic relationship between the enzymes that bind the same number of metal ions.

Although *E. coli* DHO and all other type-II DHO structures have two zinc atoms in their active sites, the type-I DHO structures vary among themselves. All of the first and second shell zinc ligands of all type I enzymes of known structure, match those of *A. aeolicus* DHO in type and position, but only the *S. aureus* enzyme (3gri.pdb) has an empty β-site like the *A. aeolicus* DHO. In contrast, the enzymes from *B. anthracis*[24] (3mpg/pdb) and *T. thermophilus* (2z00.pdb) have two active site zinc atoms, although the occupancy of the β site of the latter enzyme is only 0.4. The residues in two ligation shells around the zinc atoms in *A. aeolicus* DHO and the three other type-I DHOs were compared to understand how the metal centers of these enzymes differ (Figure 2C; see Additional file 1: Table S1).

There is precedence for variation in the metal content of the same enzymes in the amidohydrolyase superfamily. *N*-acetyl-D-galactosamine-6-phosphate deacetylase (NagA) [25] from *Bacillus subtilis* binds two metals, whereas NagA from *Escherichia coli* and *Thermotoga maritima* bind a single metal ion at the β-site. *E. coli* NagA lacks the HxH motif that forms the α-Zn site in *B. subtilis* NagA, all known type-I and type-II DHOs and many other amidohydrolases. This signature sequence is replaced by QXN and the resulting weaker metal affinity may explain why this enzyme only binds a single metal ion at the β-site [25]. A phylogenetic analysis [25] of all the NagA homologs revealed that a histidine residue located in the second ligand shell of the β-site (corresponding to His-143 in *E. coli* NagA) is conserved in all of the enzymes that cluster with the one zinc enzymes. In the two metal enzyme cluster, this residue is replaced with Glu or Gln. The authors suggest that the histidine may polarize the carbonyl bond of the substrate during hydrolysis, in effect compensating for the missing second metal ion.

However, this difference between the mono- and dinuclear enzymes is not found among the dihydroorotases. The four type-I dihydroorotases, both mono- and dinuclear, have a glycine residue (Gly154 for *A. aeolicus*) filling the space of the polarizing histidine imidazole ring, while the *E. coli* type-II DHO, has empty space. Moreover, the only sequence difference in the Zn ligation shells among the four type-I DHO structures, which occurs at position β5 in Figure 2C, does not correlate with zinc binding (Additional file 1: Table S1). The *A. aeolicus* (one zinc) and *T. thermophilus* (two zincs) enzymes have a glutamine residue at this location while *S. aureus* (one zinc) and *B. anthracis* (two zincs) enzymes have a cysteine residue.

Sequence differences in the second shell are another common explanation for variable metal affinity of histidine-rich sites. A thermodynamic analysis of zinc binding of human carbonic anhydrase provided evidence that the second shell residues, in addition to orienting the direct ligands, can also appreciably affect the enthalpy of metal binding [26]. Mutation of the second shell ligands Gln92, Glu117, and Thr199 reduces the affinity of zinc binding 10-fold [27,28]. The replacement of Glu117, which normally forms a hydrogen bond with His94 in the first shell, results in a large increase in enthalpy associated with zinc binding [27]. The structure of this mutant [28] revealed that a halide ion occupies the void created by the missing Glu side chain, but otherwise the structure of the active site is not significantly different than the wild type enzyme (C_α_ rmsd =0.2 Å).

Aspartate or glutamate residues can act as proton acceptors for the histidine residues in the first shell and thereby strengthen their interaction with the zinc atom [29,30]. This interaction is weaker when amides occupy the second shell position. However, with the type-I DHO structures, the relevant second shell ligands are the same for all four type-I DHO structures - a glutamate in α1′, an asparagine in α2′, a glutamate in β1′ and water molecules in β2′ (Figure 2C, Additional file 1: Table S1). Looking deeper, the β1′ glutamic acid is significantly farther from the β1 histidine in both *A. aeolicus* (3.3 Å) and *S. aureus* DHO (4.5 Å) - implying weaker zinc binding - than in *T. thermophilis* DHO (2.8 Å). However, the distance is just as long in *B. anthracis* DHO (3.4 Å), so again there is no simple correlation between the geometry and the relative metal affinity.

Consequently, a complete explanation of this difference in zinc affinities of the Znβ sites in the two proteins is beyond the purview of this study. For example, a recent analysis of two carbonic anhydrases had to include all atoms within 9 Å of the zinc atom in quantum mechanics calculations to fully explain the relative zinc affinities and catalytic properties [31,32].

## Conclusions

Taken together, these results indicate that although an apparent viable β-zinc binding site is formed when the *A. aeolicus* DHO domain associates tightly with ATC - presumably the natural state of these enzymes – the data presented here indicate that additional zinc does not bind to the enzyme or influence the catalytic activity. We therefore conclude that *A. aeolicus* DHO-ATC complex does not require a second metal for catalysis, although the conservation of the second site during evolution leaves open the possibility that a second ion might bind *in vivo*. However, here we have shown that this type-I DHO is fully functional with a mononuclear metal center.

## Methods

### Materials

All chemicals were purchased from Sigma-Aldrich. *E. coli* strains DH5α and BL21 (DE3) were from Invitrogen.

### Isolation of the DHO and ATC subunits and formation of the DHO-ATC complex

The genes encoding *A. aeolicus* DHO (*pyrC*) and ATC (*pyrB*) subunits were previously cloned and separately expressed in *E. coli*[5,6,33]. The genes were identified in the *A. aeolicus* genome [34], amplified by PCR and inserted into pRSETC (Invitrogen), an expression vector that incorporates a 3 kDa His tag on the amino end of the recombinant protein. The proteins were expressed individually in BL21 (DE3) and purified by Ni^+2^ affinity chromatography on a 1-ml Ni^+2^ Probond column (Invitrogen). While the DHO from some organisms has been found to have limited thermal stability [4,19], the DHO and ATC from *A. aeolicus*, a hyperthermophile that grows at 95°C [34], are stable for several months when stored in 50 mM TrisHCl, 200 mM NaCl, 200–300 mM imidazole, pH 8.0 at 4°C.

The purified subunits were mixed in an equimolar ratio to reconstitute the dodecameric DHO-ATC complex [6,12]. The complex was isolated by gel filtration chromatography on a Sephacryl S-300 column. The composition of the complex was analyzed by SDS polyacrylamide gel electrophoresis [35] and ATC and DHO assays. For crystallization, the complex was concentrated to 3 mg/ml using centrifugal filters (Amicon Ultra) with a 10 kDa molecular weight cut off. Protein concentrations were determined by the Lowry method [36] using bovine serum albumin as a standard.

### Enzyme assays

The ATC activity was measured by the colorimetric method previously described [37,38]. The reaction contained 2 mM aspartate, 5 mM carbamoyl phosphate, 1–11 μg of the purified enzyme, and 50 mM Tris–HCl, pH 8, in a total volume of 0.5 ml. The incubation time was 10 min at 37°C or 1 min at 75°C. The reactions were quenched by the addition of an equal volume of 5% acetic acid. The color was developed and quantitated as described previously [33].

DHO activity was measured in the reverse direction, the formation of carbamoyl aspartate from dihydroorotate, because the equilibrium strongly favors dihydroorotate hydrolysis [39] at pH 8.3. The formation of carbamoyl aspartate was measured using the same colorimetric method. The assay mixture, consisting of 20–45 μg DHO in 50 mM Tris acetate, pH 8.3, 10% glycerol, was preincubated for 1.5 min at 75°C. The reaction was initiated by the addition of dihydroorotate (8 mM or variable) and quenched after 1.5-5 min [6]. The kinetic parameters were obtained by least squares analysis of the dihydroorotate saturation curves using the program *KaleidaGraph*.

### Site-directed mutagenesis

Site-directed mutagenesis was carried out by PCR using Pfu Turbo polymerase (Stratagene) and the plasmid encoding the DHO subunit as a template. The putative β-site zinc ligands, His180 and His232, were replaced with alanine using forward and reverse oligonucleotides from Invitrogen. The fidelity of the constructs was verified by sequencing.

### Preparation of the metal free Apo-enzyme

Wild type DHO (25 mg) was dialyzed against 250 ml of 50 mM sodium MES, 25 mM pyridine-2,6-dicarboxylate, 1 mM EDTA, 30% glycerol (v/v), 50 mM sodium sulfate, 0.5 mM DTT, pH 5.5, with two changes of the dialysis buffer over three days [6]. The apoenzyme was concentrated and desalted by passage over a Sephadex G-25 column equilibrated with 20 mM MES, 20% glycerol 0.1 mM TCEP, pH 6.3. The protein was then concentrated to 10 mg/ml. The complete removal of zinc was verified by zinc spectrophotometric analysis and the DHO-ATC complex was reconstituted by mixing equimolar amounts of the metal-free DHO and ATC subunits.

### Metal analysis

The zinc content was measured by determining the absorbance of the complex of zinc with 4-(2-pyridylazo)resorcinol (PAR), in 50 mM HEPES, pH 7.4 [40]. The DHO-ATC (10 μM) was denatured in 4 M guanidine HCl for 15 minutes and PAR was added to a final concentration of 50 μM. The absorbance was read at 497 nm, the λ_max_ of Zn-PAR (ϵ_497_ = 46,700 M^-1^cm^-1^). The zinc released from the protein was calculated using a ZnCl_2_ standard curve (0–20 μM). Alternatively, the zinc and cobalt composition of the protein was analyzed by inductively coupled plasma (ICP) mass spectrometry. The purified proteins in 50 mM TrisHCl, 200 mM NaCl and 2 mM DTT were supplemented where indicated with 100 μM ZnCl_2_. The excess zinc was removed by gel filtration on a G25 column in the same buffer. The final protein concentration was approximately 40 μM as determined by the Lowry method [36]. The samples were acidified by the addition of HNO_3_ to 2% and analyzed in a Perkin Elmer Sciex Elan 9000 ICP-MS with a cross flow nebulizer and Scott type spray chamber. The RF power was 1000 and the argon flow was set at 0.92 L/min. Analytical grade standard metal stock solutions were purchased from Sigma-Aldrich and VWR.

### Crystallization of the DHO-ATC mutant

Before crystallization, the protein was buffer exchanged into 10 mM HEPES, 1 mM TCEP, pH 7.5, at a final protein concentration of 3.0 mg/ml. All crystals were grown at room temperature using the hanging-drop, vapor-diffusion technique: 3–6 μl of the DHO-ATC solution was mixed with 1 μl of reservoir solution (30% ethylene glycol) followed by 10% of the drop volume (v/v) of 100 mM barium chloride as a crystallization additive [12]. Prism-like crystals appeared within 2–7 days; the best diffracting crystals grew to 0.2 × 0.2 × 0.1 mm and were often accompanied by small needle-like crystals. Prior to data collection, the crystals were soaked overnight in the cryo solution: 10 mM BaCl_2_, 35% v/v ethylene glycol, 1 mM PALA and 15 mM dihydroorotate at pH 5.

### Phasing and refinement of the DHO-ATC double mutant

The X-ray diffraction data were collected in 360 frames at the Advanced Photon Source (IMCA-CAT) using a wavelength of 0.97856 Å and a rotation of 0.75 degree per frame. The data were integrated with iMosfilm [41] and truncated at 2.2 Å where the average intensity in the highest shell was approximately twice the estimated error. The initial electron density map was calculated using phases of the native DHO-ATC [12], refit with ARP/wARP [42] to reduce bias, and refined with REFMAC5 [43]. All solvent density was initially fit and refined with water molecules. Strong solvent peaks with anomalous diffraction density were refit with barium ions. Clusters of 4 water molecules at unusually short distances from each other were refit with molecules of ethylene glycol. The final ratio of *R*_free_/*R* was 1.26, which is very close to the expected value of 1.25 at this resolution [44]. There are no residues in the disallowed regions of the Ramachandran plot calculated by PROCHECK.

### Modeling and analysis

Unless stated otherwise, structures compared in this paper were superposed with SSM [45], which includes secondary structure matches in the calculation. SPDBV [46] was used to overlap structures based upon specific residues. To facilitate comparison of the active sites, a virtual second zinc site (Zn1006) was modeled into the structure of *S. aureus* DHO (3gri.pdb; Zn500) by overlapping the DHO structure from *T. thermophilis* (2z00.pdb; Zn1004, Zn1006) using only the main chain atoms of the α1, α2, and αβ4 residues (Figure 2C). This approach placed the respective Znα atoms (Zn500, Zn1004) within 0.2 Å of each other and the transformed Znβ atom (Zn1006) 3.2 Å from the native Znα atom (Zn500) in the *S. aureus* structure. Panels showing crystallographic structures were prepared with Pymol [47].

### Availability of supporting data

The data set supporting the results of this article is included in the article and its Additional file 1: Table S1.

## Endnotes

^a^The structure of mammalian DHO was presented by Santiago Ramon-Maiques at the 23rd International Conference on Arginine and Pyrimidines held at the Universidad de los Andes, Bogotá, Colombia. The manuscript is pending publication. A preliminary X-ray analysis of the crystals has been published [48].

^b^The atomic coordinates for the crystal structure discussed herein will be available from the Research Collaboratory for Structural Bioinformatics with accession code 4BJH upon publication.

## Abbreviations

AQUAE: *Aquifex aeolicus*; ATC: Aspartate transcarbamoylase; BACAN: *Bacillus anthracis*; CAD: A polypeptide chain present in mammals and some other species that includes the first three enzymes of pyrimidine biosynthesis fused in the order, CPS–DHO–ATC; CPS: Carbamoyl phosphate synthetase; DAC: Dihydroorotase-aspartate transcarbamoylase complex; DHO: Dihydrorotase; DOR: Dihydroorotate; PALA or PALN: (Phosphonacetyl)-L-aspartate; PAR: 4-(2-pyridylazo)resorcinol; STACC: *Staphylococcus aureus*; THETA8: *Thermus thermophilus.*

## Competing interests

The authors declare that they have no competing interests.

## Author’s contributions

RF, EG and MC carried out the metal analysis and kinetic studies; RF also constructed the DHO mutants; AV isolated the proteins, reconstituted the DHO ATC complex, conducted the ICP metal analysis and grew the protein crystals; JB collected the X-ray crystallography data and carried out the initial analysis of the data; PM solved the structure of the mutant complex; BFPE refined and analyzed the final, submitted structure; BFPE, HE and DRE were primarily responsible for the experimental design, interpretation of the data and writing the manuscript. All authors read and approved of the final manuscript.

## Supplementary Material

Additional file 1: Table S1 Interaction parameters for the first and second ligation shells of Znα and Znβ depicted in Figure 1C. Click here for file
