# Supplementary material for: The mononuclear metal center of type-I dihydroorotase from aquifex aeolicus
Source: BMC Biochem. 2013 Dec 9;14:36. doi: 10.1186/1471-2091-14-36 (PMC3880350; doi:10.1186/1471-2091-14-36)
Supplement: Additional file 1: Table S1 — Interaction parameters for the first and second ligation shells of Znα and Znβ depicted in Figure 1C. [file 1471-2091-14-36-S1.docx]

TABLE S1: Interaction parameters for the first and second ligation shells of Znα and Znβ depicted in Figure 1C.

| **DHO^3^** | **AQUAE** | **AQUAE** | **STAAC** | **STAAC** | **BACAN** | **BACAN** | **THET8** | **THET8** |
| --- | --- | --- | --- | --- | --- | --- | --- | --- |
| **PDB** | **3D6N**  **Partner** | **Distance (Å)** | **3GRI**  **Partner** | **Distance (Å)** | **3MPG**  **Partner** | **Distance (Å)** | **2Z00**  **Partner** | **Distance (Å)** |
|  |  |  |  |  |  |  |  |  |
| **Znα** | Zn423 |  | ZN500 |  | Zn429 |  | Zn1004 |  |
| **Znβ** | J577 | 4.0 | (Zn1006)^2^ | 3.2 | Zn430 | 3.3 | Zn1006 | 3.3 |
| SHELL#1 |  | Zn - #1 |  |  |  |  |  |  |
| **α1** | H61 | 2.3 | H58 | 2.1 | H59 | 2.2 | H55 | 2.1 |
| **α2** | H63 | 2.2 | H60 | 2.5 | H61 | 2.4 | H57 | 2.2 |
| **α3** | D153 | 2.4 | D150  J417 | 4.7  2.3 | D151 | 2.5 | D147 | 2.2 |
| **α4** | D305 | 2.7 | D303 | 2.3 | D304 | 2.4 | D306 | 2.4 |
| **α5** | FLC | 2.1 | J491 | 2.4 | NIL | – | J1125 | 2.4 |
|  |  |  |  |  |  |  |  |  |
| **β1** | H180 | 2.4 | H177 | 2.2 | H178 | 2.4 | H174 | 2.2 |
| **β2** | H232 | 2.2 | H230 | 2.2 | H231 | 2.4 | H233 | 2.4 |
| **β3** | D153 | 2.3 | D150  J417 | 4.15  3.20 | D151 | 2.5 | D147 | 2.1 |
| **β4** | D305 | 5.2 | D303 | 4.7 | D304 | 4.9 | D306 | 4.6 |
| **β5** | FLC  Q231 | 2.65  3.39 | J491  C229 | 3.81  5.73 | NIL  C230 | –  5.56 | J1125  Q232 | 3.23  4.94 |
| SHELL#2 |  | #1 - #2 |  |  |  |  |  |  |
| **α1'** | E255 | 2.6 | E253 | 3.0 | E254  J504 | 3.1  3.3 | E256 | 2.8 |
| **α2'** | N95 | 2.7 | N92 | 3.0 | N93 | 2.8 | N89 | 2.9 |
| **α3'** | H61  H63 | 3.3  3.3 | NIL | NIL | H59  H61 | 3.3  2.9 | H55  H57 | 2.7  3.4 |
| **α4'** | H61  FLC  FLC | 3.1  2.9  3.1 | H58  J491 | 3.0  2.9 | NIL | – | H55  J1125 | 3.0  2.7 |
| **α5'** | D305  D305  H63 | 2.9  3.1  3.5 | D303  H60  J154  J244 | 2.9  2.9  3.1  3.5 | NIL | – | D306  A279  J1007 | 2.7  2.6  2.9 |
|  |  |  |  |  |  |  |  |  |
| **β1'** | E182  J560 | 3.3  2.9 | E179  N276  J231 | 4.5  3.6  4.0 | E180 | 3.4 | E176 | 2.8 |
| **β2'** | J630 | 3.3 | J231 | 3.4 | NIL | – | J1043  J1054 | 3.4  3.5 |
| **β3'** | NIL | – | J417  J367  G151 | 3.1  3.0  3.1 | NIL | – | Q232 | 3.6 |
| **β4'** | FLC  J510  J551 | 3.1  3.2  2.3 | J186  J161  J244 | 2.6  3.3  3.5 | NIL | – | J1010  J1125  J1031 | 3.3  2.7  2.5 |
| **β5'** | H180 | 3.2 | same as a5' | – | NIL | – | same as a5' |  |

^2^Uniprot identifiers for the organism and specific strain: AQUAE (*A. aeolicus*), STAAC (*S. aureus*), BACAN (*B. anthracis*), THET8 (*T. thermophilus*).

^3^Modeled from *T. thermophiles* DHO Znβ as described in the methods section.
